# Management of toxic optic neuropathy via a combination of Wharton’s jelly-derived mesenchymal stem cells with electromagnetic stimulation

**DOI:** 10.1186/s13287-021-02577-2

**Published:** 2021-09-27

**Authors:** Emin Özmert, Umut Arslan

**Affiliations:** 1 Department of Ophthalmology, Faculty of Medicine, Ankara University, Ankara, Turkey; 2 Ankara University Technopolis, Bioretina Eye Clinic, Neorama Ofis 55-56 Yaşam Cad. No 13/A, Beştepe /Yenimahalle, Ankara, Turkey

**Keywords:** Toxic optic neuropathy, Methanol, Sildenafil, Amiodarone, Carbon dioxide, Stem cell, Wharton’s jelly-derived mesenchymal stem cell, Electromagnetic stimulation

## Abstract

**Purpose:**

To investigate the effect of the combination of Wharton's jelly derived mesenchymal stem cells (WJ-MSC) and high frequency repetitive electromagnetic stimulation (rEMS) in the therapy of toxic optic neuropathies with severe symptoms after the available current therapy modalities which were unsucessful.

**Material and methods:**

This prospective, open-label clinical phase-3 study was conducted at Ankara University Faculty of Medicine, Department of Ophthalmology between April 2019 and April 2021. Thirty-six eyes of 18 patients with toxic optic neuropathy (TON) were included in the study. Within 1–3 months after the emergency interventions, patients with various degrees of sequela visual disturbances were studied in this clinical trial. The cases were divided into three groups according to similar demographic characteristics. Group 1: Consists of 12 eyes of 12 patients treated with the WJ-MSC and rEMS combination in one eye. Group 2: Consists of 12 eyes of 12 patients treated with only rEMS in one eye. Group 3: Consists of 12 eyes of six patients treated with only WJ-MSC in both eyes. The course was evaluated by comparing the quantitive functional and structural assessment parameters measured before and at the fourth month of applications in each group.

**Results:**

The mean best corrected visual acuity (BCVA) delta change percentages of the groups can be ranked as: Group 1 (47%) > Group 3 (32%) > Group 2 (21%). The mean fundus perimetry deviation index (FPDI) delta change percentages of the groups can be ranked as: Group 1 (95%) > Group 2 (33%) > Group 3 (27%). The mean ganglion cell complex (GCC) thickness delta change (decrease in thickness) percentages can be ranked as: Group 1 (− 21%) > Group 3 (− 15%) > Group 2 (− 13%). The visual evoked potential (VEP) P100 latency delta change percentages of the groups can be ranked as: Group 1 (− 18%) > Group 3 (− 10%) > Group 2 (− 8%). The P100 amplitude delta change percentages of the groups can be ranked as: Group 1 (105%) > Group 3 (83%) > Group 2 (24%).

**Conclusion:**

Toxic optic neuropathies are emergent pathologies that can result in acute and permanent blindness. After poisoning with toxic substances, progressive apoptosis continues in optic nerve axons and ganglion cells. After the proper first systemic intervention in intensive care clinic, the WJ-MSC and rEMS combination seems very effective in the short-term period in cases with TON. To prevent permanent blindness, a combination of WJ-MSC and rEMS application as soon as possible may increase the chance of success in currently untreatable cases.

*Trial Registration* ClinicalTrials.gov ID: NCT04877067.

## Introduction

The visual function begins with converting light energy into biochemical, electrical signals in the outer layers of the retina. Electrical signals are transmitted from the photoreceptors (first neuron) to the bipolar cells (second neuron) and then to the ganglion cells (third neuron). The axons of the ganglion cells (retinal nerve fibers) form the optic nerve. The number of optic nerve axons from the post-laminar region to the lateral geniculate nucleus is approximately 1,200,000. The optic nerve transmits electrical signals to the visual center located in the brain’s occipital cortex through various synapses, including the lateral geniculate nucleus (fourth neuron) [1–3]. Optic nerve fibers are more sensitive to various toxins than the retina because they are outside the protecting feature of the blood-retinal barrier. Methanol, various solvents and heavy metals, carbon dioxide, antiarrhythmic and antiepileptic drugs, some antibiotics, and vasoactive drugs can cause toxic optic neuropathy (TON). There is different pathophysiology for each toxic substance resulting in optic nerve damage. Metabolites of some toxins disrupt adenosine three phosphate (ATP) synthesis by blocking mitochondrial function and oxidative phosphorylation. Metabolites of some other toxins cause demyelination as a result of protein denaturation. Neuroinflammation occurs when denatured proteins block axoplasmic flow. Neuroinflammation also develops secondary to the cessation of axoplasmic flow after hypoxia. Hypoxic neurons stop their metabolism and switch to OFF mode. If hypoxia and neuroinflammation persist, apoptosis and permanent vision loss develop [4, 5].

Wharton’s jelly-derived mesenchymal stem cells (WJ-MSC) can increase mitochondrial ATP synthesis via various growth factors (GF) and suppress neuroinflammation with an immunomodulatory effect [6–10]. High frequency repetitive electromagnetic stimulation (rEMS) can rearrange ion channel balances and axoplasmic flow. The effects of rEMS are also known to increase blood flow and synaptic transmission in neural tissues, decrease toxic glutamine levels and increasing the passage of large therapeutic molecules into the cell [11–14].

The aim of this prospective clinical study is to investigate the effect of the combined use of WJ-MSC and rEMS in the management of TON after the interventions in emergency or intensive care units. This combination therapy is the first study in the literature to treat TON with severe symptoms, in which available current therapy modalities were unsucessful.

## Materials and methods

Ethics committee approval for the umbilical cord Wharton’s jelly-derived mesenchymal stem cell (WJ-MSC) study was obtained from the Ankara University Faculty of Medicine Clinical Research Ethics Committee (19-1293-18). It was also approved by the Review Board of the Cell, Organ, and Tissue Transplantation Department within the Turkish Ministry of Health (56733164/203 E.1925). Ethics committee approval for the transcranial electromagnetic stimulation study was obtained from the Ankara University Faculty of Medicine Clinical Research Ethics Committee (17-1177-18) and the Review Board of the Drug and Medical Device Department within the Turkish Ministry of Health (2018-136). The study was performed following the tenets of the 2013 Declaration of Helsinki. Written informed consent was obtained from the patients before enrollment.

This prospective, open-label clinical phase-3 study was conducted at Ankara University Faculty of Medicine, Department of Ophthalmology between April 2019 and April 2021. Thirty-six eyes of 18 patients with toxic optic neuropathy (TON) were included in the study. The primary toxic optic neuropathy (TON) diagnosis of the affected patients was made in an emergency or intensive care clinic. Within 1–3 months after the emergency interventions, patients with various degrees of sequela visual disturbances were studied in this clinical trial. All patients enrolled underwent a complete routine ophthalmic examination, including the best-corrected visual acuity (BCVA) measurement with the early treatment of diabetic retinopathy study (ETDRS) chart (Topcon CC 100 XP, Japan). The patients were further evaluated with optical coherence tomography angiography (OCTA) from RTVue XR (Avanti, Optovue, Fremont, CA, USA), which provides a co-registered en-face and cross-sectional multimodal imaging platform to analyze and measure the changes in the ganglion cell layer (GCL). Functional evaluation of optic nerve was made by Compass 24/2 visual field (VF) test (Compass, CenterVue, Padova, Italy) and the 120-pattern visual evoked potential (pVEP) test (Mon 2018F, Metrovision, Perenchies, France). Before the different treatment modalities with WJ-MSC and rEMS and at the fourth month after the treatments, quantitative assessment parameters were compared.

### Subjects

Thirty-six eyes of 18 patients with toxic optic neuropathy (TON) due to four different kinds of toxic substances (mainly methanol, CO_2_, sildenafil, amiodaron) were included in the clinical study. Different ophthalmic therapy modalities (only WJ-MSC, only rEMS or both) were applied to the cases between 1 to 3 months after discharge from hospital, so as to eliminate the possible therapeutic effect of the medical treatment done for intoxication, and before the development of irreversible optic nerve damage, which might occur after 3 months.

The inclusion criteria were: The patients who could be assessed by quantitative parameters of aforementioned tests at baseline (just before the treatment) and at fourth month after the treatment; patients with best-corrected visual acuity (BCVA) better than 35 letters, for performing appropriate visual field testing; any degree of visual field loss; and patients over 18 years old.

The exclusion criteria were: Cases poisoned with toxic substances for more than 3 months; patients with BCVA less than 35 letters, in whom visual field test can not be done properly; non-cooperated patients because of neurological sequelae; previous history of diabetes mellitus and cardiovascular diseases; and smokers.

#### Study groups

Thirty-six eyes of 18 patients exposed to four types of toxic substances composed the study cohort. The eyes could be divided into three groups according to the applied treatment modalities with similar demographic characteristics.

In 12 patients with TON, due to ethical considerations, the worse eye received one subtenon injection of WJ-MSC. Ten days after the injection, rEMS was applied on both eyes of these patients for 30 min via a custom-designed helmet. rEMS applications were repeated ten times with a 1-week interval during the trial. So the 12 eyes of the patients received both WJ-MSC and rEMS constitute Group 1, and other 12 eyes of the same patients received only rEMS constitute Group 2. Another different 6 patients’ both eyes (total 12 eyes) with TON received only one subtenon injection of WJ-MSC without rEMS application, which form Group 3.

The course was evaluated by comparing the BCVA, FDPI, GCC thickness, pVEP-p100 latency, and amplitude parameters measured before and at the fourth month of applications in each group (Tables 1, 2, 3).

**Table 1:** Group1: Demonstration of demographic characteristics, structural and functional changes of group1 to which WJ-MSC and rEMS combination was applied

Patient no	Eye	Toxin	BCVA		Visual field FPDI		GCC thickness		VEP P100 lat.VEP P100 lat		VEP P100 ampl	
			Before	After	Before	After	Before	After	Before	After	Before	After
1	L	Methanol	80	98	49	69	101	82	132	105	3.1	5.8
2	R	Methanol	35	92	15	72	92	64	148	112	2.1	4.7
3	L	Amiodarn	89	110	34	98	103	88	136	101	3.4	7.2
4	L	Methanol	35	80	47	65	88	61	154	114	1.1	4.2
5	R	Sildenafil	50	83	38	61	94	60	144	114	1.8	3.4
6	L	Methanol	50	98	37	59	91	70	139	106	2.9	6.3
7	L	C0_2_	39	65	11	28	71	52	140	118	3.6	7.1
8	R	Methanol	35	45	7	14	62	50	149	131	2.7	3.9
9	R	Methanol	40	50	5	11	64	51	157	136	1.4	2.6
10	R	Methanol	54	54	8	10	62	58	147	128	1.6	2.9
11	L	Methanol	35	40	4	11	58	56	145	130	1.0	2.6
12	R	Methanol	35	35	1	2	55	50	153	132	1.2	2.3

*WJ-MSC* Wharton’s jelly derived mesenchymal stem cell, *rEMS* repetitive electromagnetic stimulation, *BCVA* best corrected visual acuity (ETDRS letters), *FPDI* fundus perimetry deviation index (%), *GCC thickness* Ganglion cell complex (µm), *VEP* visual evoked potential, *P100 lat* latency (ms), *P100 ampl* amplitude (mV)

**Table 2:** Group2: Demonstration of demographic characteristics, structural and functional changes of group2 to which only rEMS was applied

Patient no	Eye	Toxin	BCVA		Visual Field FPDI		GCC Thickness		VEP P100 lat		VEP P100 ampl	
			Before	After	Before	After	Before	After	Before	After	Before	After
1	R	Methanol	80	89	61	71	103	91	129	114	3.2	4.7
2	L	Methanol	35	80	13	42	88	78	144	126	2.2	3.4
3	R	Amiodaron	90	100	49	72	112	108	131	110	3.5	4.1
4	R	Methanol	35	50	46	53	74	58	156	128	1.0	2.1
5	L	Sildenafil	60	74	42	56	89	64	141	119	2.0	2.9
6	R	Methanol	50	74	40	52	88	74	137	120	3.0	4.1
7	L	CO_2_	40	50	13	17	74	59	138	124	3.7	4.3
8	L	Methanol	35	35	12	12	57	52	144	141	2.9	2.9
9	L	Methanol	45	45	7	8	65	53	154	155	1.7	1.6
10	L	Methanol	59	59	8	8	62	59	146	145	1.6	1.4
11	R	Methanol	36	36	3	2	58	57	153	150	1.7	1.7
12	L	Methanol	39	39	2	2	56	54	152	151	1.3	1.3

*rEMS* repetitive electromagnetic stimulation, *BCVA* best corrected visual acuity (ETDRS letters), *FPDI* fundus perimetry deviation index (%), *GCC thickness* Ganglion cell complex (µm), *VEP* visual evoked potential, *P100 lat* latency (ms), *P100 ampl* amplitude (mV)

**Table 3:** Group3: Demonstration of demographic characteristics, structural and functional changes of group3 to which only WJ-MSC was applied

Patient no	Eye	Toxin	BCVA		Visual field FPDI		GCC thickness		VEP P100 lat		VEP P100 ampl	
			Before	After	Before	After	Before	After	Before	After	Before	After
1	R	Methanol	65	85	61	78	89	76	141	121	2.9	4.8
2	L	Methanol	60	80	56	68	82	73	145	123	2.7	3.8
3	R	Sildenafil	35	60	28	36	68	59	151	129	1.4	2.6
4	L	Sildenafil	40	70	32	37	72	63	146	119	1.8	2.9
5	R	Methanol	35	40	7	12	68	56	159	153	1.5	3.1
6	L	Methanol	35	45	10	15	71	58	152	152	1.6	6.5
7	R	Methanol	35	35	14	18	66	57	153	151	1.2	2.7
8	L	Methanol	45	60	21	27	69	59	149	141	1.7	3.2
9	R	Methanol	50	65	31	39	81	70	144	131	2.1	3.6
10	L	Methanol	54	70	33	41	86	75	140	121	2.6	3.9
11	R	Methanol	65	80	41	52	98	81	138	119	3.1	4.9
12	L	Methanol	60	74	39	49	94	80	140	121	2.9	4.6

*WJ-MSC* Wharton’s jelly derived mesenchymal stem cell, *BCVA* best corrected visual acuity (ETDRS letters), *FPDI* fundus perimetry deviation index (%), *GCC thickness* Ganglion cell complex (µm), *VEP* Visual evoked potential, *P100 lat* latency (ms), *P100 ampl* amplitude (mV)

#### Group 1

Consists of 12 eyes of 12 patients treated with the WJ-MSC and rEMS combination in one eye. WJ-MSC was applied first to the patients after necessary preparations. rEMS application was started 10 days after the WJ-MSC application. The rEMS was applied with a custom-designed helmet for 30 min after the subtenon WJ-MSC application. WJ-MSC was applied only one time. rEMS applications were repeated ten times with a 1-week interval. The course was evaluated by comparing the BCVA, FDPI, GCC thickness, pVEP-p100 latency, and amplitude parameters measured before and fourth month of applications (Table 1).

#### Group 2

Consists of 12 eyes of 12 patients treated with only rEMS in one eye. rEMS was applied with a custom-designed helmet for 30 min. rEMS applications were repeated ten times with 1-week intervals. The course was evaluated by comparing the BCVA, FDPI, GCC thickness, pVEP-p100 latency, and amplitude parameters measured before and fourth month of applications (Table 2).

#### Group 3

Consists of 12 eyes of six patients treated with only WJ-MSC in two eyes. Only WJ-MSC was applied to the patients after necessary preparations. WJ-MSC was applied only one time for both eyes. The course was evaluated by comparing the BCVA, FDPI, GCC thickness, pVEP-p100 latency, and amplitude parameters measured before and in the fourth month of applications (Table 3).

### Umbilical cord Wharton’s jelly-derived mesenchymal stem cells preparation

**Fig. 1:**
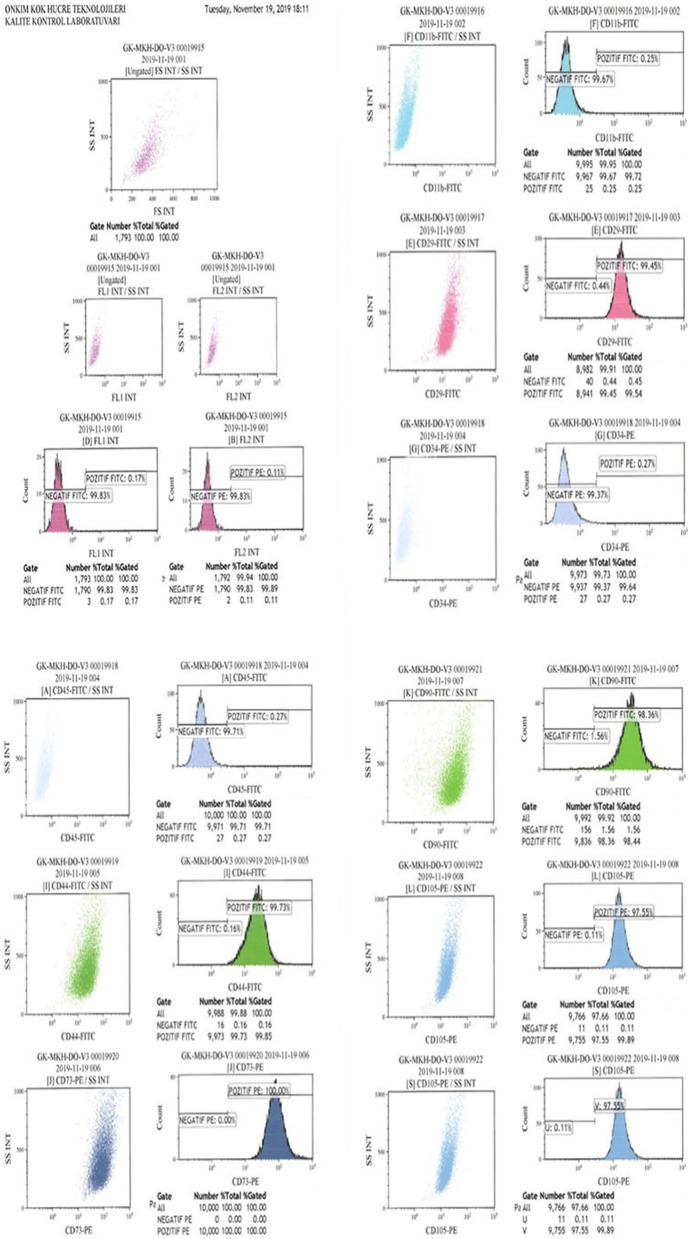

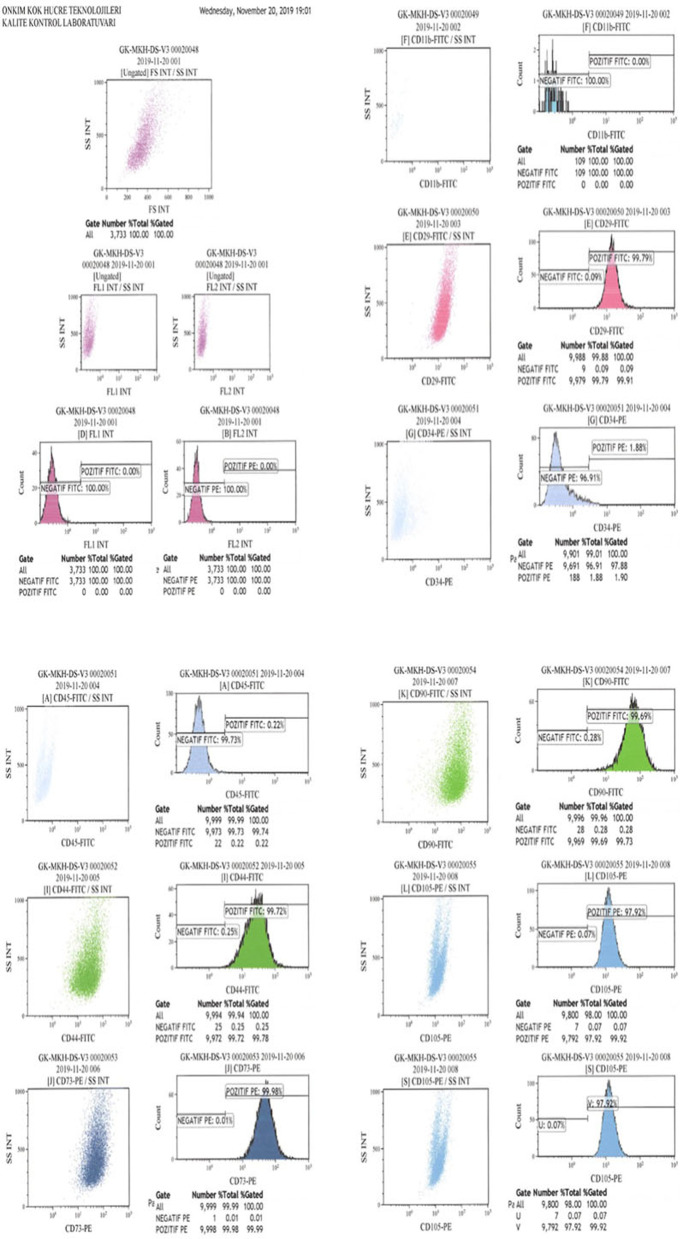
**a** The phenotypic characterization of Wharton jelly derived mesenchymal stem cells before cryopreservation. **b** The phenotypic characterization of Wharton jelly derived mesenchymal stem cells after cryopreservation

The mesenchymal stem cells used in this study were isolated from Wharton’s jelly of the umbilical cord collected allogenicly from a single donor with the mother’s consent. The umbilical cord sample was treated following several steps. Briefly, cord tissue was washed twice with phosphate-buffered saline (Lonza, Switzerland), and the Wharton’s jelly part was minced using forceps and a scalpel. Minced pieces were cultivated in a cell culture dish (Greiner Bio-One, Germany) with Dulbecco’s modified Eagle’s medium F12 (DMEM)-low glucose with no L-glutamine (Biological Industries, Israel) and 10% human AB serum (Capricorn, Germany), 1% 10.000 U/mL penicillin, and 10.000 μg/mL streptomycin (Gibco, USA). All cell preparations and cultivation procedures were conducted in a current Good Manufacturing Practice (cGMP) accredited laboratory (Onkim Stem Cell Technologies, Turkey). The culture-expanded cells were cryopreserved at P3 using standard cryopreservation protocols until used in the following experiment. CryoSure-DEX40 (WAK-Chemie Medical, Germany) containing 55% Dimethyl Sulfoxide and 5% Dextran 40 was used as cryopreservant. The cells were characterized at the time of cryopreservation using flow cytometric analysis to determine the expression of the positive cluster of differentiation (CD) surface markers, CD90, CD105, CD73, CD44, CD29, and negative for CD34, CD45, and CD11b (Fig. 1a, b). Using real-time polymerase chain reaction (qPCR), the expressions of several genes, such as tumor necrosis alpha (*TNF alpha*) and vimentin (*VIM*), were analyzed. Additionally, quality control analyses, such as mycoplasma and endotoxin analyses (using the PCR and LAL test combined with sterility analysis, respectively) were also completed. Cells were solubilized from cryopreservation before being prepared for injection. The average cell viability for each treatment was over 90.0%, and each patient received 2–6 × 10^6^ cells in a 1.5 ml saline solution (Fig. 1a, b).

### Injection of umbilical cord WJ-MSCs

The WJ-MSCs suspension from the culture was delivered to the operating room by cold chain and used within 24 h. A total of 1.5 ml of the WJ-MSC suspension was immediately injected into the subtenon space of each eye. The procedure was conducted under topical anesthesia with proparacaine hydrochloride drops (Alcaine, Alcon, USA) and sterile conditions. A 5/0 atraumatic traction suture was applied to the limbus for easy access and manipulation to the application area. A small cut was made through the conjunctiva and tenon capsule up to the sclera in the superior-temporal quadrant, 13 mm away from the limbus, to insert a 20 G subtenon curved cannula (BD, Visitec, UK). Subsequently, a 7/0 vicryl suture was passed through the conjunctiva and tenon and tied down with a loop creation. A curved subtenon cannula attached to the 2.5 cc syringe filled with 1.5 ml fluid containing stem cells was inserted through the cut and forwarded into the extraocular muscle conus until reaching the sclera. Fluid (1.5 ml) was then injected. While the cannula was drawn back, a loop was tightened to prevent leakage. Postoperatively, loteprednol and tobramycin combination eye drops were given four times per day for 1 week, and oral amoxicillin-clavulanate (1 g) was given twice a day for 5 days.

### Retinal repetitive electromagnetic stimulation (rEMS)

**Fig. 2:**
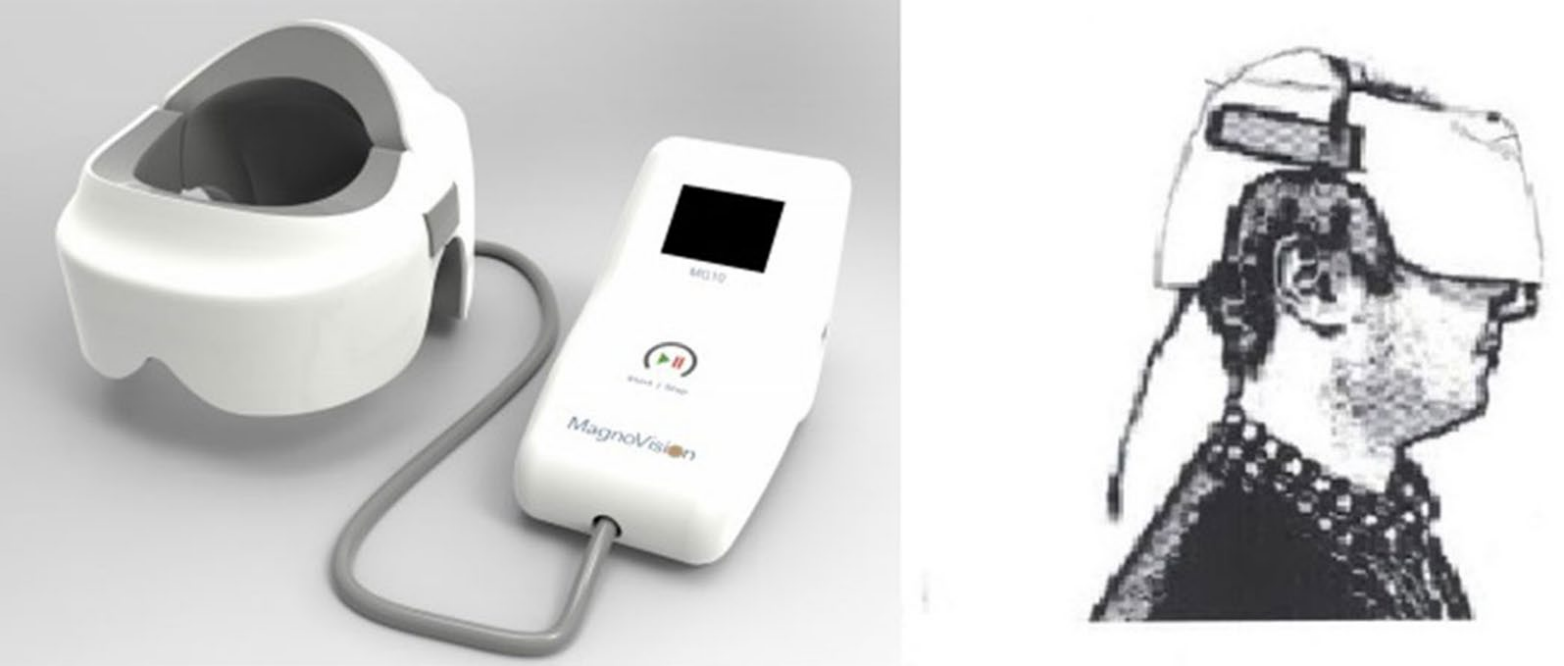
Retinal electromagnetic stimulator (rEMS) device. Application of the helmet to stimulate the retina-optic nerve and visual pathways [35–37]

Specifically designed helmet producing repetitive high-frequency electromagnetic stimulation (rEMS) Magnovision™, Bioretina Biotechnology, Ankara, Turkey) stimulated the retinas and visual pathways in both eyes with an electromagnetic field strength of 2000 miligauss, frequency of 42 Hz, and duration of 30 min. These values were previously determined to be effective for other clinical and preclinical studies (Fig. 2).

### Timeframe

The patients were evaluated at several study timepoints:

- **T0**: Baseline evaluation; to evaluate the structural and functional conditions of the eyes due to toxicity just before the treatment modalities
- **T1**: First-month assessment; for clinical/ophthalmoscopic evaluation and possible complications. Quantitative parameters were not studied.
- **T2**: Fourth-month assessment; structural and functional evaluation to assess the value of treatment modalities. An ophthalmic examination was made to detect possible complications.

### Primary outcome measure

ETDRS visual acuity: The visual acuity scores obtained from the T0 and T2 examinations were analyzed and compared using statistical tests to determine effectiveness.

*Secondary outcome measures*: The following scores obtained from the T0 and T2 examinations were analyzed and compared using statistical tests to determine effectiveness.

*Visual field sensitivity*: Fundus perimetry deviation index (FPDI, %).

FPDI was examined in the 24/2 visual field of the computerized perimetry records. The FPDI offers data explaining how many of the 100 flashing points and what percentage of the visual field could be correctly seen by the patient. For VF analysis, practice rounds were carried out three times before applications to avoid mistakes during the test.

*Ganglion cell complex thickness (GCC thickness, µm)*: GCC is the thickness from the internal limiting membrane to the inner plexiform layer in the 3 × 3 mm of foveal area. The measurement is done automatically by the OCTA device. GCC is the total thickness of the ganglion cells and retinal nerve fibers (OCTA from RTVue XR, Avanti, Optovue, Fremont, CA, USA).

*Pattern visual evoked potential (pVEP)*: pVEP is an objective test that measures the electrical activity of the optical pathway in response to a light stimulus. The 120 patterns reveal responses from all retinal quadrants. The measurements were taken according to the ISCEV standards for both eyes. We used the 120-pattern VEP protocol, which combines p100 implicit time and amplitude, to create a numerical result.

### Definition of safety outcome

Orbital inflammation, diplopia, ocular allergic reactions, intraocular hemorrhages, retinal vessel occlusions, retinal detachment, and acute glaucoma were serious adverse ocular events. B-scan orbital ultrasonography was also used to detect and confirm complications at T1 and T2 time points. Systemic allergic reactions and anaphylaxis were considered to be systemic side effects.

### Statistical methods

Descriptive statistics are presented with frequency, percentage, mean, and standard deviation values. The Kruskal Wallis test was used to analyze the differences in BCVA, FPDI, GCC thickness, pVEP P100 amplitude, and implicit time scores according to the T0 and T2 times. The Mann–Whitney U test was used for measurement differences between groups. The Sidak test was used to compare delta changes between groups. In the study, *p *-values < 0.05 were considered statistically significant (*α* = 0.05). Analyses were done with the SPSS 25.0 package program.

## Results

*The mean age and type of toxicity*: The mean age was 39.9 years (range, 22–58 years) in Group 1 (10 male, two female); 39.9 years (range, 22–58 years) in Group 2 (10 male, two female); and 38.3 years (range, 26–59 years) in Group 3 (12 male). There was no statistical difference between the groups in terms of age (*p* = 0.63). In Group 1, methanol was the main cause of intoxication in nine cases, amiodarone in one case, sildenafil in one case, and CO_2_ in one case. Group 2 consisted of the fellow eyes of the patients in Group 1, which is why the reasons for intoxication were the same. Group 3 consisted of 12 eyes of six patients, and methanol was the main cause of intoxication in ten cases and sildenafil in two cases (Tables 1, 2, 3). Overall, methanol toxicity was the main reason seen in 77.7% of cases.

*The mean best-corrected visual acuity (m-BCVA)*: Group 1 could identify a mean of 48.1 letters before with WJ-MSC + rEMS applications and mean 70.8 letters after the procedures at fourth month (*p* = 0.01). Group 2 had an m-BCVA of 50.3 letters at baseline and 60.9 letters after rEMS applications at the fourth month (*p* = 0.03). Group 3 had an m-BCVA score of 48.3 letters before WJ-MSC applications and 63.7 letters after the applications in the fourth month (*p* = 0.02). The BCVA delta change percentages of the groups can be ranked as: Group 1 (47%) > Group 3 (32%) > Group 2 (21%) (Tables 1, 2, 3,4, 5).

**Table 4:** Statistical comparison of measurements according to groups

Measurements	Group			pG1	pG2	pG3
	Group1	Group2	Group3			
	*X* ± s.s	*X* ± s.s	*X* ± s.s			
BCVA before	48.08 ± 18.44	50.33 ± 18.58	48.25 ± 12.26	0.01*	0.03*	0.02*
BCVA after	70.83 ± 25.84	60.92 ± 21.93	63.67 ± 16.33			
Visual field FPDI before	21.33 ± 18.14	24.67 ± 21.12	31.08 ± 16.81	0.01*	0.02*	0.04*
Visual field FPDI after	41.67 ± 32.29	32.92 ± 27.31	39.33 ± 20.31			
GCC thickness	78.42 ± 17.99	77.17 ± 18.77	78.67 ± 11.16	0.01*	0.04*	0.02*
GCC thickness	61.83 ± 12.47	67.25 ± 17.41	67.25 ± 9.54			
VEP P100 lat. before	145.33 ± 7.56	143.75 ± 8.95	146.5 ± 6.4	0.01*	0.03*	0.02*
VEP P100 lat. after	118.92 ± 12.03	131.92 ± 15.67	131.75 ± 13.73			
VEP P100 ampl before	2.16 ± 0.94	2.32 ± 0.91	2.13 ± 0.68	0.01*	0.04*	0.01*
VEP P100 ampl after	4.42 ± 1.79	2.88 ± 1.24	3.88 ± 1.14			

**Mann Whitney *U* test, *p < 0.05: statistically signifficant

*BCVA* best corrected visual acuity, (ETDRS letters), *FPDI* fundus perimetry deviation index (%), *GCC thickness* Ganglion cell complex (µm), *VEP* Visual evoked potential, *P100 lat* latency (ms), *P100 ampl* amplitude (mV)

**Table 5:** Statistical comparison of delta changes (Δ) according to groups

Measurements	Group			*p* comparision
	Group1	Group2	Group3	
	Δ	Δ	Δ	
BCVA	47% ^G1^	21%^G2^	32%^G3^	0.01* G1 > G3 > G2
Visual field FPDI	95%^G1^	33%^G2^	27%^G3^	0.01* G1 > G2 > G3
GCC thickness	− 21%^G1^	− 13%^G2^	− 15%^G3^	0.01* G1 > G3 > G2
VEP P100 lat	− 18%^G1^	− 8%^G2^	− 10%^G3^	0.01* G1 > G3 > G2
VEP P100 ampl	105%^G1^	24%^G2^	83%^G3^	0.01* G1 > G3 > G2

Δ rate of change was calculated as the last-first measurement / first measurement. **(Sidak comparision test, *p < 0.05: statistically signifficant)

*BCVA* best corrected visual acuity (ETDRS letters), *FPDI* fundus perimetry deviation index (%), *GCC thickness* Ganglion cell complex (µm), *VEP* visual evoked potential, *P100 lat* latency (ms), *P100 ampl* amplitude (mV)

**Fig. 3:**
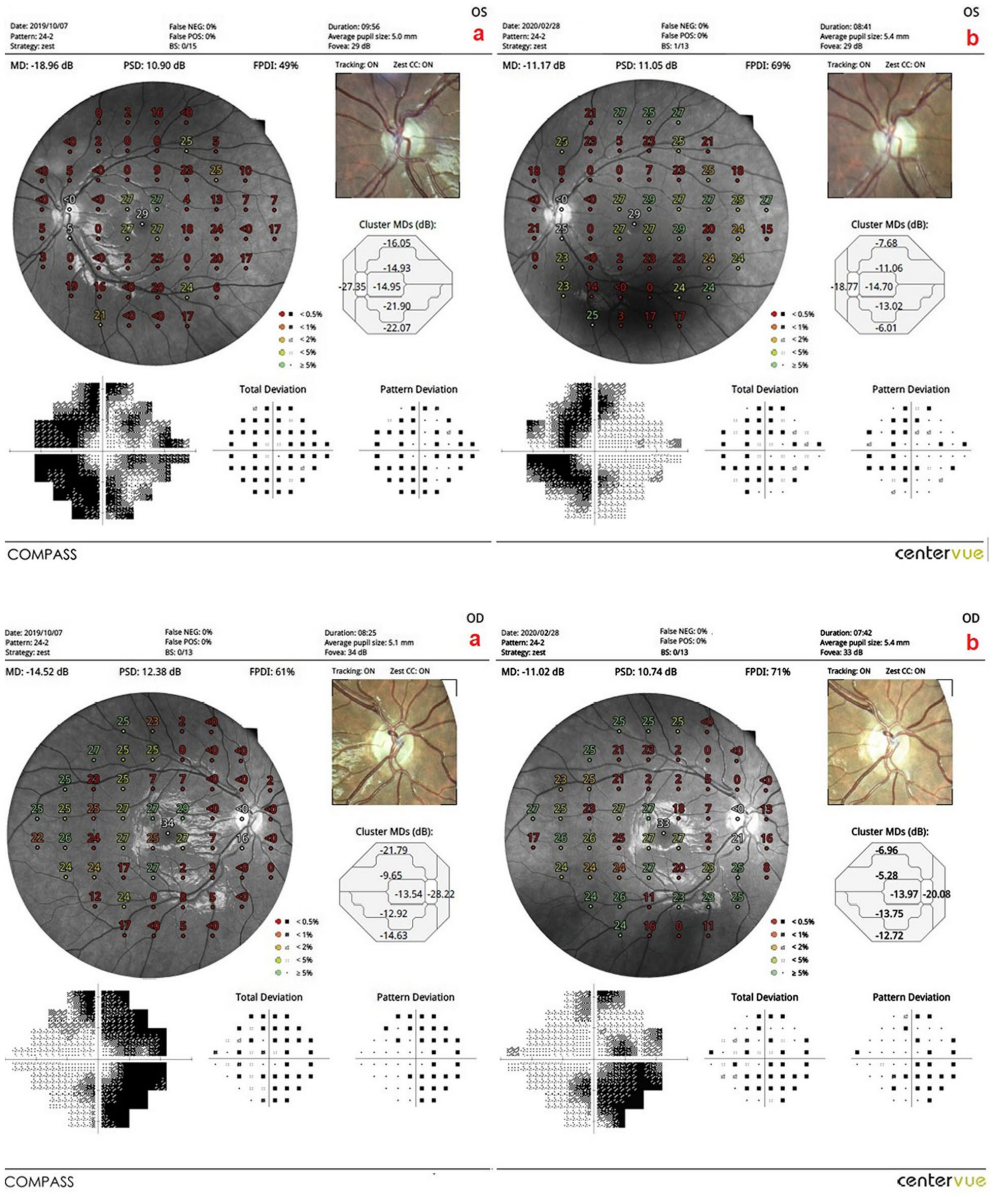
**a** Visual field enlargement according to study timepoints (T0, T2) in the eye treated with combination of WJ-MSC and rEMS. Note the change in FPDI values (Table 1, patient 1: left eye). (a**)** Before application, FPDI 49% (b) at 4th month, FPDI 69%. **b** Visual field enlargement according to study timepoints (T0, T2) in the eye treated with only rEMS. Note the change in FPDI values (Table 2, patient 1: right eye). (a) Before application, FPDI 61% (b) at 4th month, FPDI 71%

**Fig. 4:**
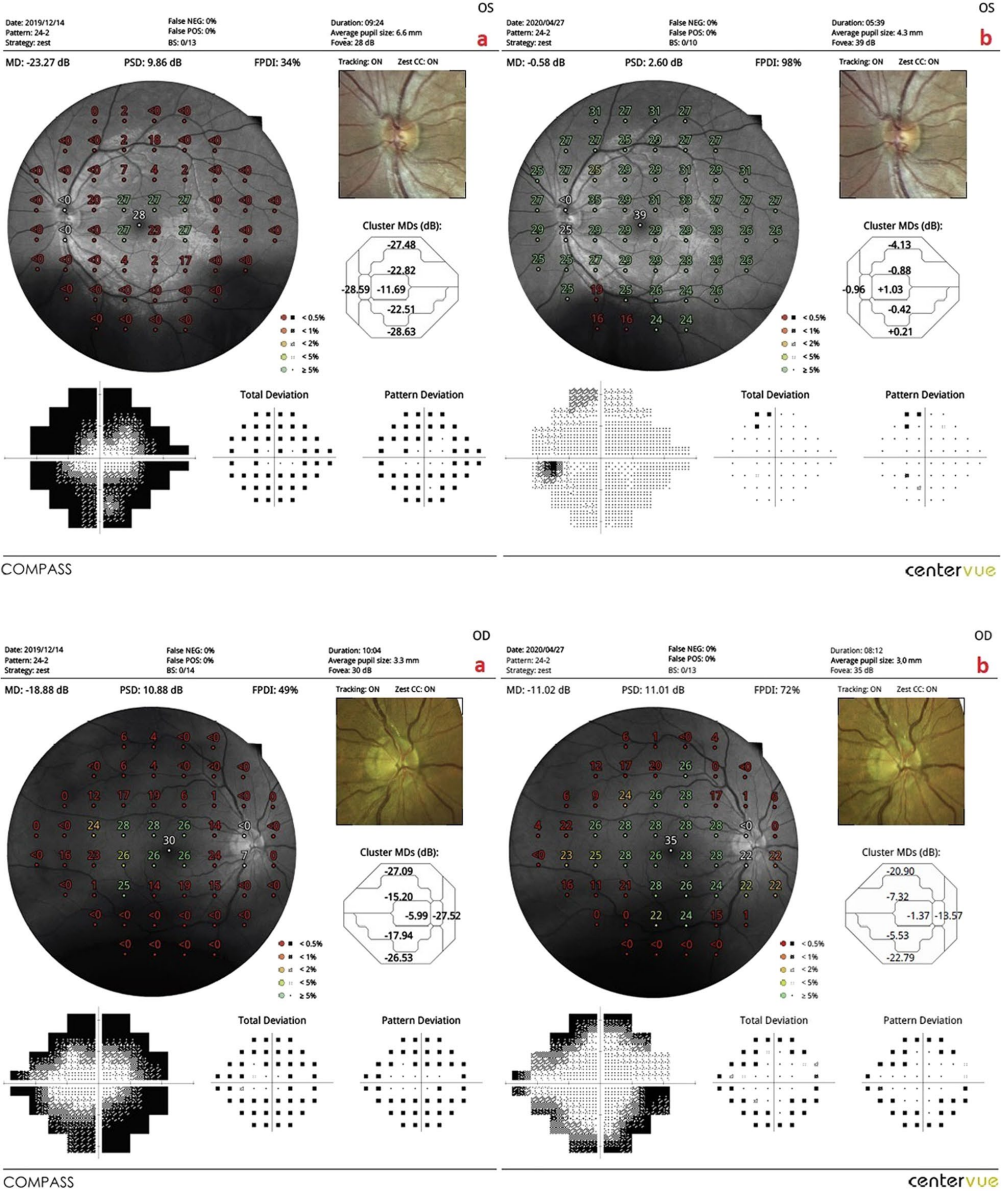
**a** Visual field enlargement according to study timepoints (T0, T2) in the eye treated with combination of WJ-MSC and rEMS. Note the change in FPDI values (Table 1, patient 3: left eye). (a) Before application, FPDI 34% (b) at 4th month, FPDI 98%. **b** Visual field enlargement according to study timepoints (T0, T2) in the eye treated with only rEMS. Note the change in FPDI values (Table 2, patient 3: right eye). (a) Before application, FPDI 49% (b) at 4th month, FPDI 72%

**Fig. 5:**
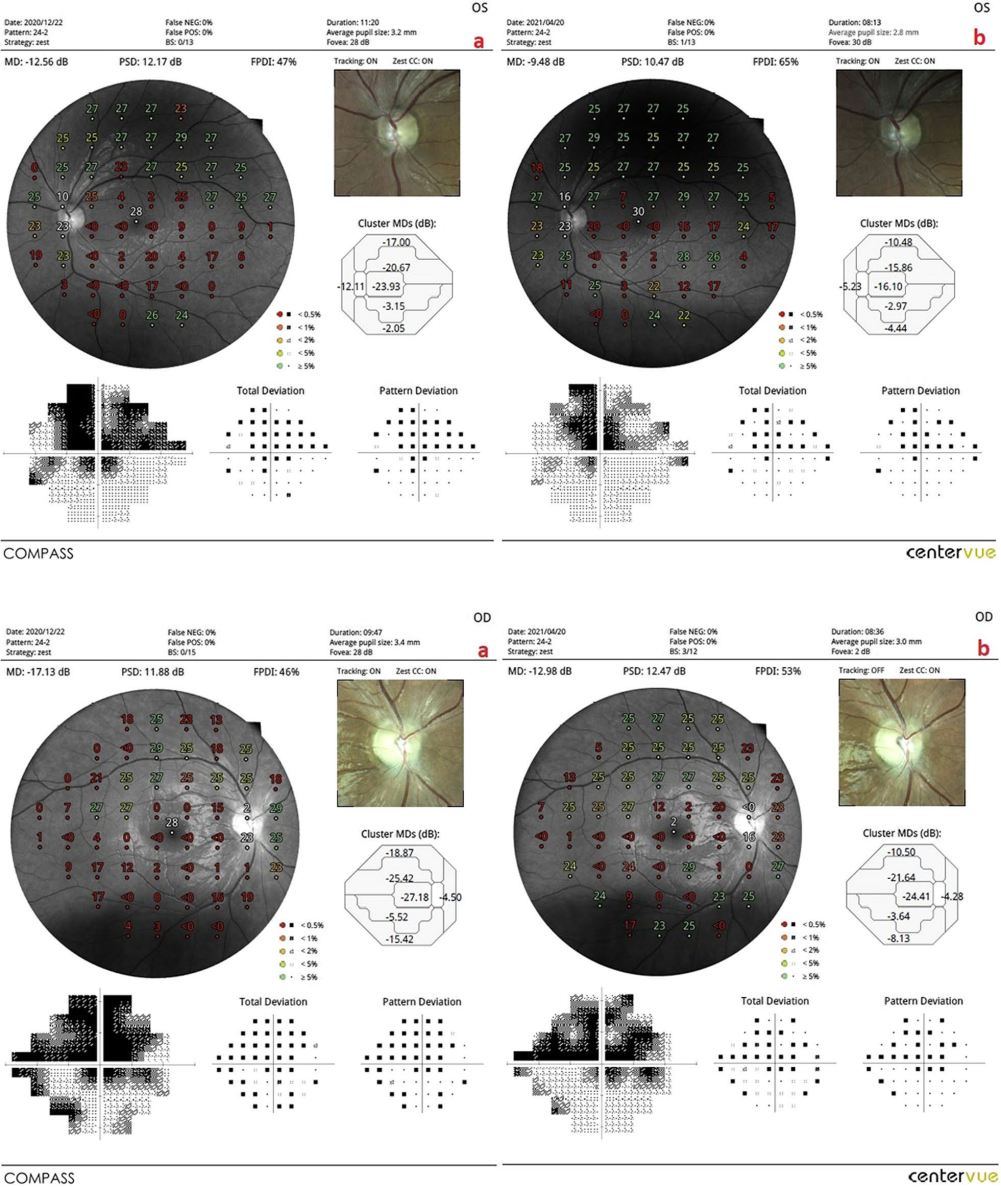
**a** Visual field enlargement according to study timepoints (T0, T2) in the eye treated with combination of WJ-MSC and rEMS. Note the change in FPDI values (Table 1, patient 4: left eye). (a) Before application, FPDI 47% (b) at 4th month, FPDI 65%. **b** Visual field enlargement according to study timepoints (T0, T2) in the eye treated with only rEMS. Note the change in FPDI values (Table 2, patient 4: right eye). (a) Before application, FPDI 46% (b) at 4th month, FPDI 53%

**Fig. 6:**
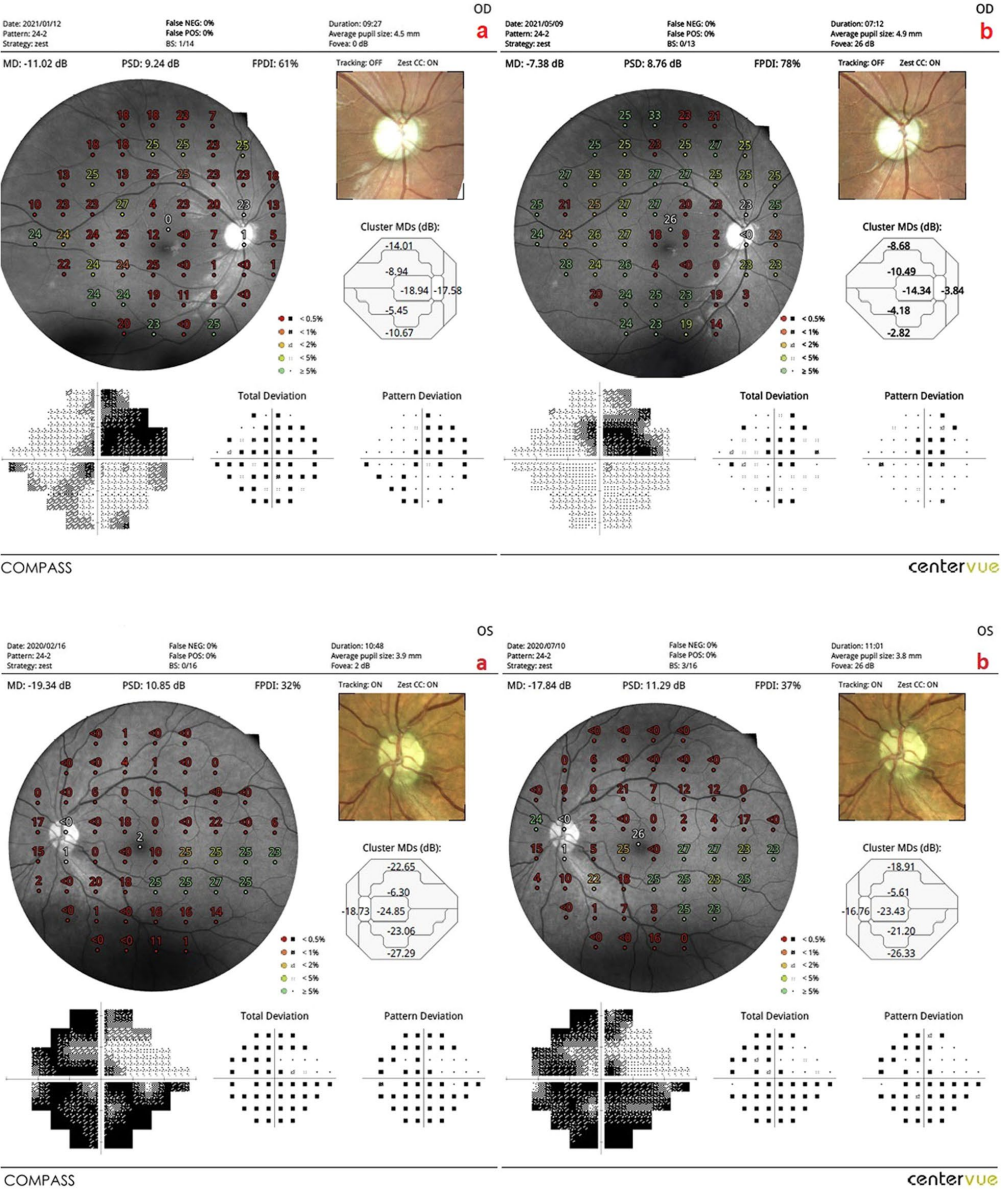
**a** Visual field enlargement according to study timepoints (T0, T2) in the eye treated with only WJ-MSC. Note the change in FPDI values (Table 3, patient 1: right eye). (a) Before application, FPDI 61% (b) at 4th month, FPDI 78%. **b** Visual field enlargement according to study timepoints (T0, T2) in the eye treated with only WJ-MSC. Note the change in FPDI values (Table 3, patient 4: left eye). (a) Before application, FPDI 32% (b) at 4th month, FPDI 37%

*The mean of the fundus perimetry deviation index (m-FDPI)*: This value was 21.3% in Group 1 before the combined WJ-MSC and rEMS applications and 41.7% after the procedures at the fourth month (*p* = 0.01). In Group 2, the m-FDPI was 24.7% at the first measurement and 32.9% after only rEMS applications at the fourth month (*p* = 0.02). In Group 3, the m-FDPI was 31.1% before WJ-MSC applications and 39.3% at the last examination in the fourth month (*p* = 0.04). The m-FPDI delta change percentages of the groups can be ranked as: Group 1 (95%) > Group 2 (33%) > Group 3 (27%) (Tables 1, 2, 3, 4, 5; Figs. 3, 4, 5, 6).

**Fig. 7:**
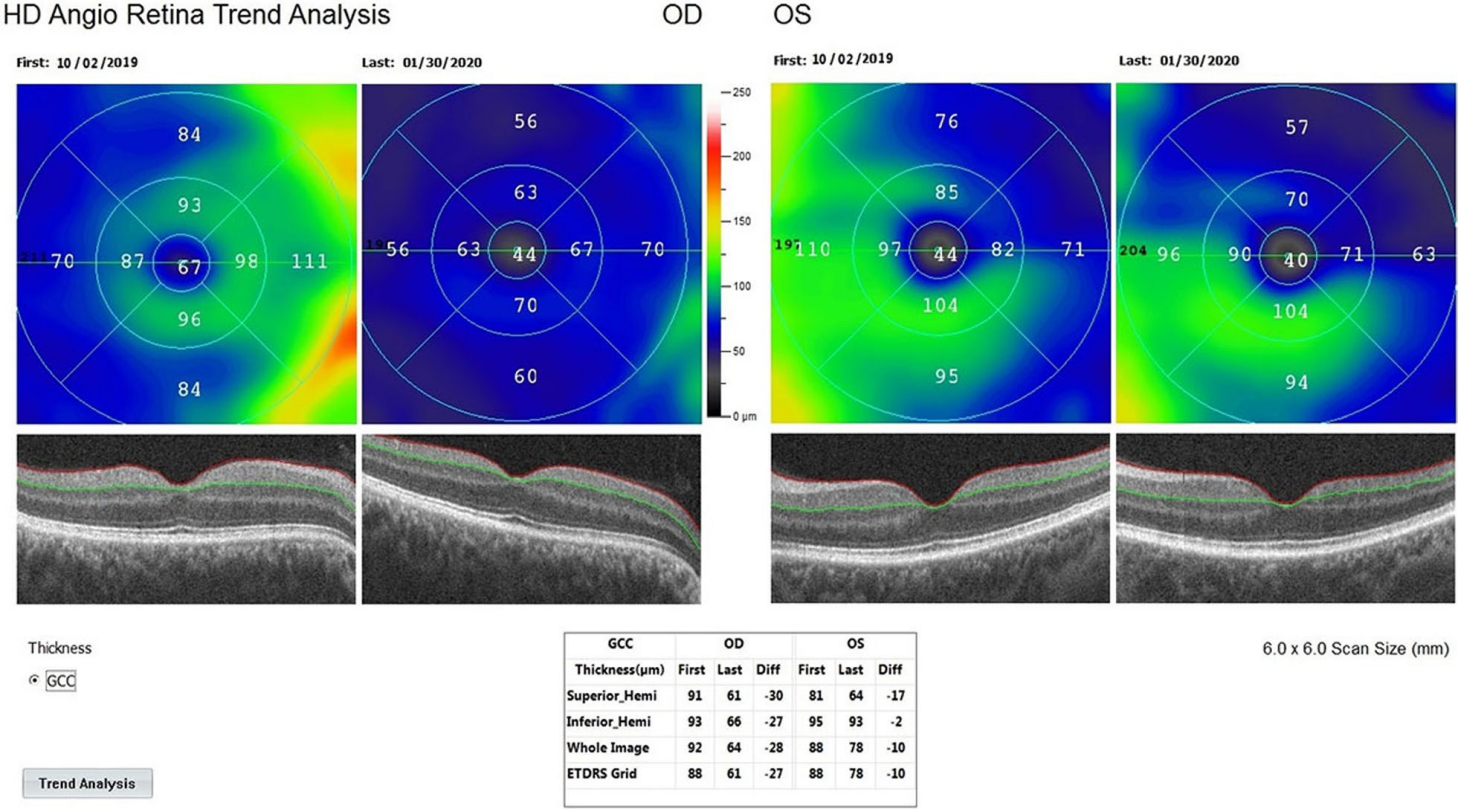
Improvement in “ganglion cell complex thickness” according to study timepoints (T0, T2) in the OD eye treated with combination of WJ-MSC and rEMS (Table 1, patient 2: right eye. (a) Before application, 92 µm, (b) at 4th month, 64 µm. Improvement in “ganglion cell complex thickness” according to study timepoints (T0, T2) in the OS eye treated with only rEMS (Table 2, patient 2: left eye. (a) Before application, 88 µm, (b) at 4th month, 78 µm

**Fig. 8:**
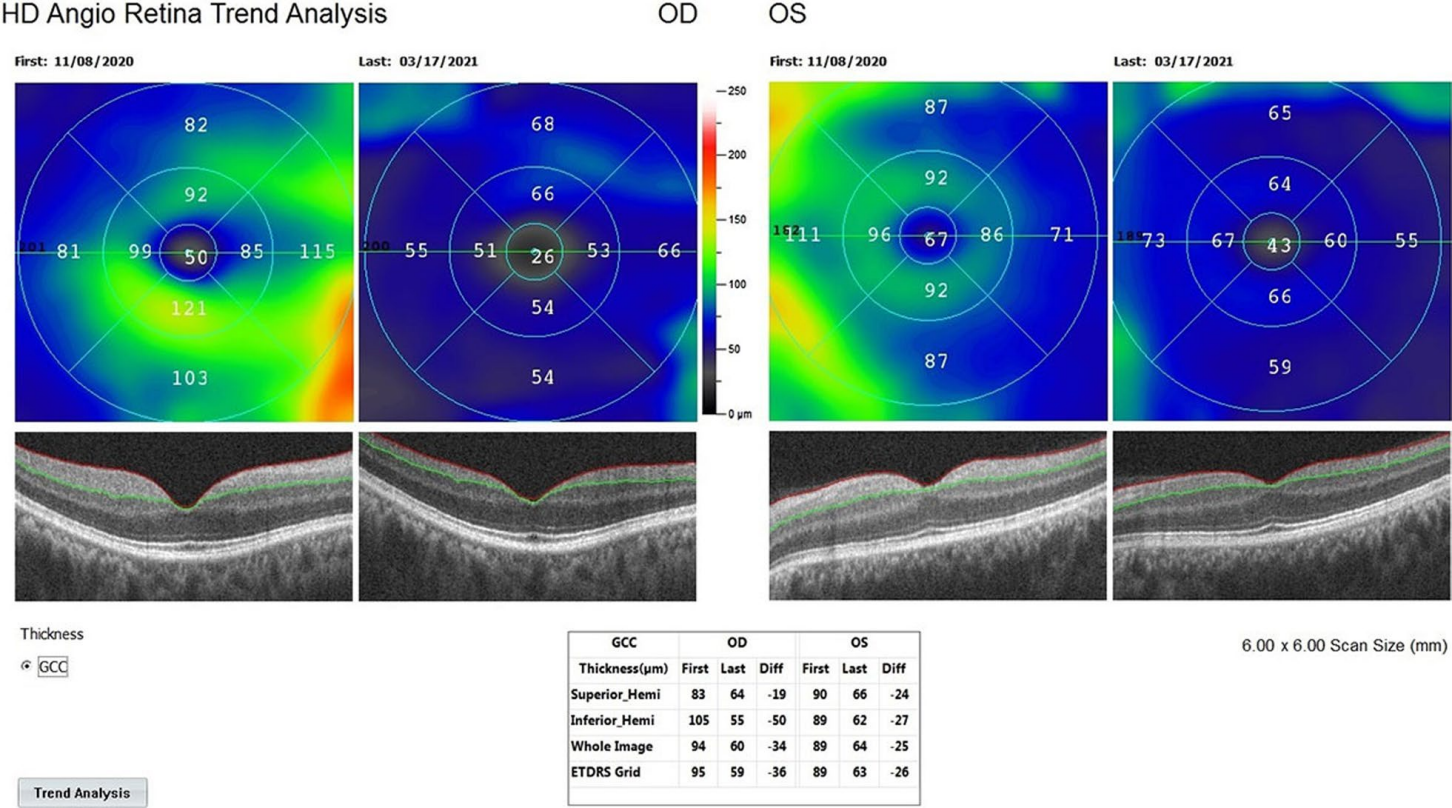
Improvement in “ganglion cell complex thickness” according to study timepoints (T0, T2) in the OD eye treated with combination of WJ-MSC and rEMS (Table 1, patient 5: right eye. (a) Before application, 94 µm, (b) at 4th month, 60 µm. Improvement in “ganglion cell complex thickness” according to study timepoints (T0, T2) in the OS eye treated with only rEMS (Table 2, patient 5: left eye. (a) Before application, 89 µm, (b) at 4th month, 64 µm

*The mean ganglion cell complex (m-GCC) thickness* in Group 1 was 78.4 μm before combined management and 61.8 μm after the procedures (*p * = 0.01). In Group 2, the m-GCC thickness was 77.2 μm at the first and 67.3 μm after the rEMS applications (*p * = 0.04). In Group 3, the m-GCC thickness was 78.7 μm before WJ-MSC applications and 67.3 μm after the applications (*p * = 0.02). The m-GCC thickness delta change (decrease in thickness) percentages can be ranked as: Group 1 (− 21%) > Group 3 (− 15%) > Group 2 (− 13%) (Tables 1, 2, 3, 4, 5; Figs. 7 and 8).

**Fig. 9:**
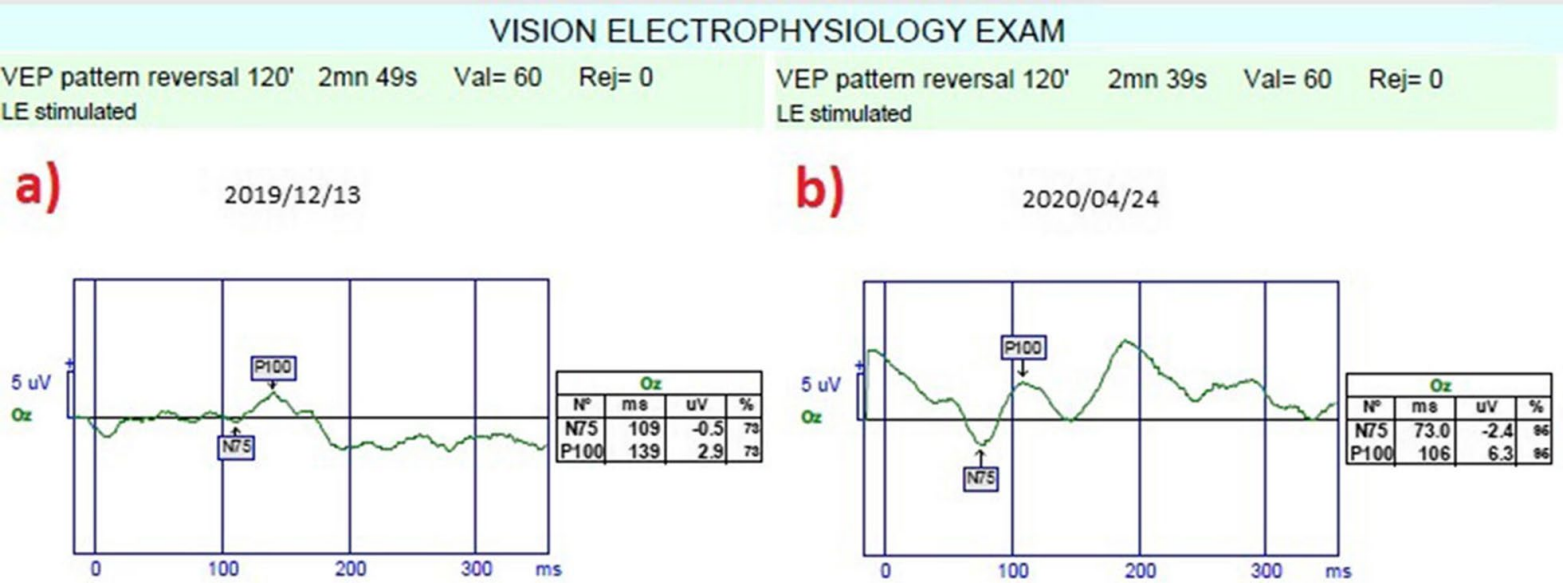
Improvement in “pattern VEP” according to study timepoints (T0, T2) in the eye treated with combination of WJ-MSC and rEMS (Table 1, patient 6: left eye). **a** Before application, P100 latency 139 ms, P100 amplitude 2.9 mV **b** at 4th month, P100 latency 106 ms, P100 amplitude 6.3 mV

**Fig. 10:**
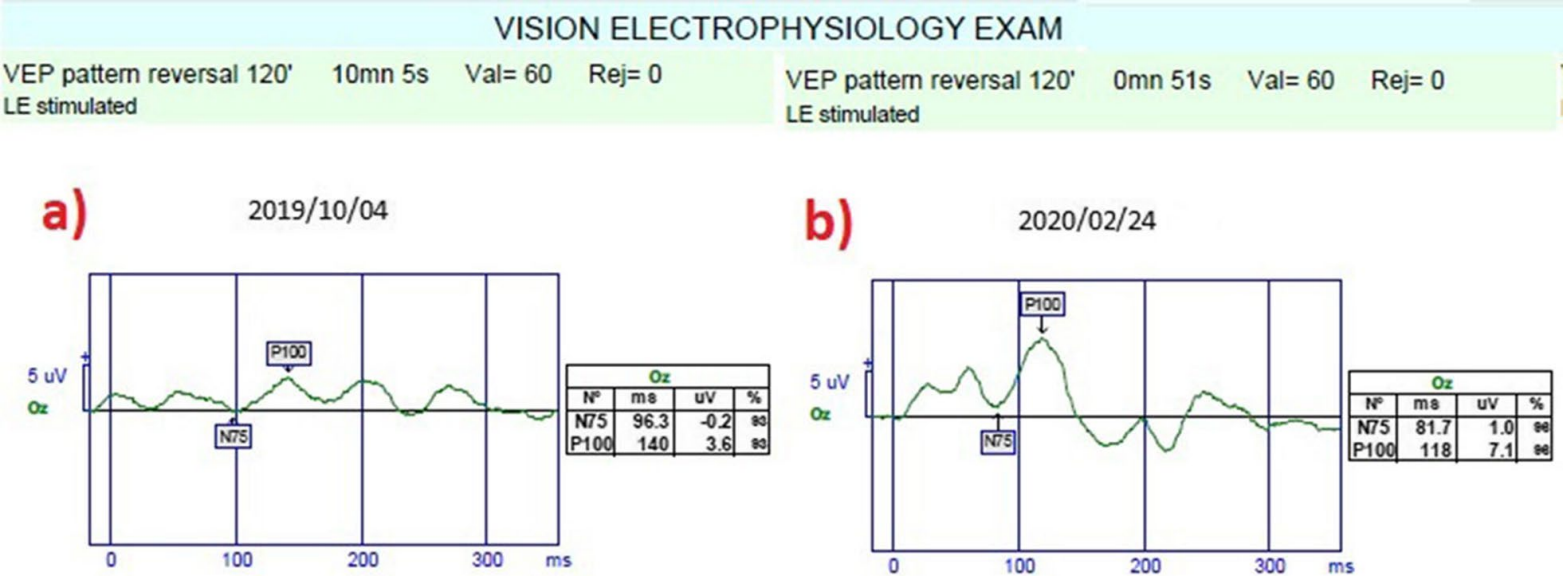
Improvement in “pattern VEP” according to study timepoints (T0, T2) in the eye treated with combination of WJ-MSC and rEMS (Table 1, patient 7: left eye). **a** Before application, P100 latency 140 ms, P100 amplitude 3.6 mV **b** at 4th month, P100 latency 118 ms, P100 amplitude 7.1 mV

**Fig. 11:**
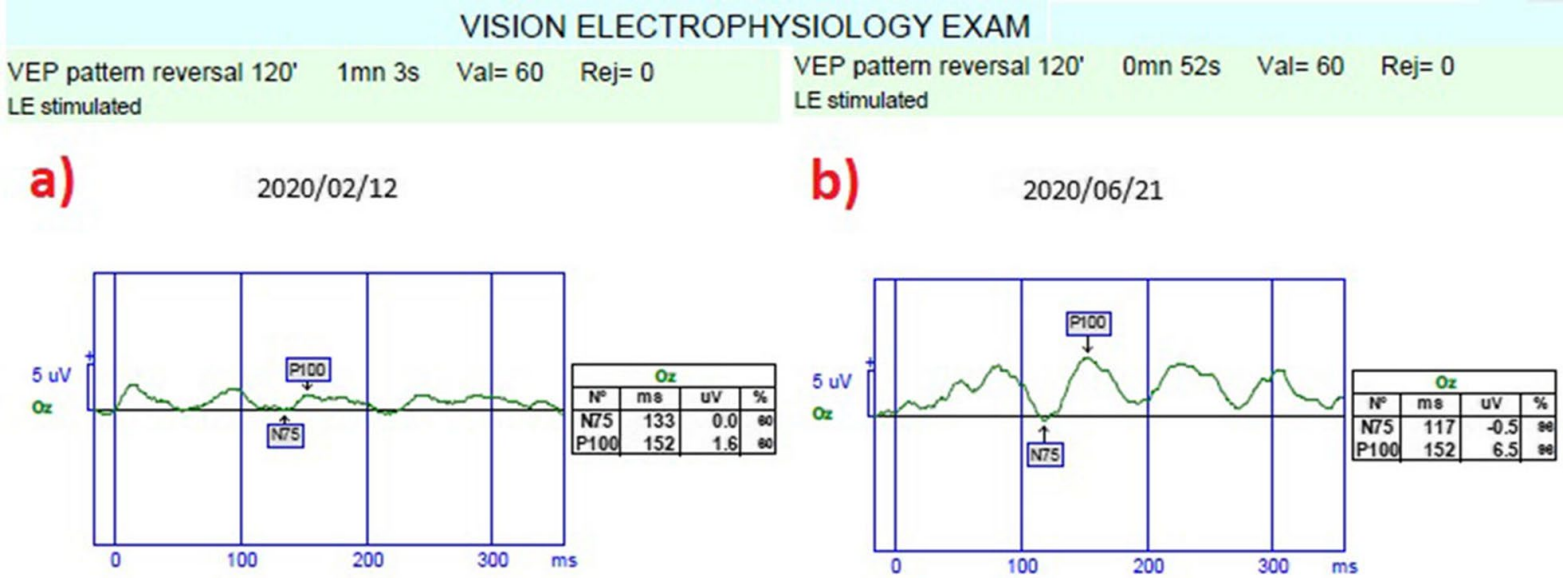
Improvement in “pattern VEP” according to study timepoints (T0, T2) in the eye treated with only WJ-MSC (Table 3, patient 6: left eye). **a** Before application, P100 latency 152 ms, P100 amplitude 1.6 mV **b** at 4th month, P100 latency 152 ms, P100 amplitude 6.5 mV

*The mean pattern visual evoked potentials P100 (m-P100) amplitudes and latency*: The mean P100 latency was 145.3 m in Group 1 before the combined application and 118.9 ms after the procedures (*p * = 0.01). In Group 2, the P100 latency was 143.8 ms at the first measurement and 131.9 ms after rEMS applications (*p * = 0.03). In Group 3, the mean P100 latency was 146.5 ms before WJ-MSC applications and 131.8 ms at the last examination (*p * = 0.02). The P100 latency delta change percentages of the groups can be ranked as: Group 1 (− 18%) > Group 3 (− 10%) > Group 2 (− 8%). The mean P100 amplitude was 2.2 mV in Group 1 before the combined application and 4.4 mV after the procedures (*p * = 0.01). In Group 2, the P100 amplitude was 2.3 mV at the first and 2.9 mV after rEMS applications (p*p *= 0.04). In Group 3, the mean P100 amplitude was 2.1 mV before WJ-MSC applications and 3.9 mV at the last examination (*p * = 0.01). The P100 amplitude delta change percentages of the groups can be ranked as: Group 1 (105%) > Group 3 (83%) > Group 2 (24%) (Tables 1, 2, 3, 4, 5; Figs. 9, 10, 11).

When Groups 1, 2, and 3 were compared using the Sidak test according to the delta change percentages, the combined application of WJ-MSC and rEMS significantly increases all assessment parameters (Table 5).

No serious ocular or systemic adverse events were encountered in any group related to WJ-MSC and rEMS applications in the fourth month. The patients are still being followed for the assessment of long-term results.

## Discussion

The axons of the ganglion cells, which form the retinal nerve fiber layer, are unmyelinated until the lamina cribrosa. Retinal nerve fibers are composed of the optic nerve head in the prelaminar region. Microtubules form the skeleton of the axons, and retinal nerve fibers are surrounded by oligodendrocytes and become myelinated in the post-laminar region. Organelles, mitochondria, protein synthesis, intracellular digestion, and all vital activities take place in ganglion cells. Bidirectional axoplasmic flow occurs for the vital and functional activities of axons. From the ganglion cells to the lateral geniculate nucleus, structural and functional proteins, neurotransmitters in vesicles, mitochondria, and ions flow towards the synaptic end. Neurotransmitters, organelles, and ions need to be regenerated, and cellular wastes need to be digested to flow from the synaptic end to the ganglion cells [15–18]. Oligodendrocytes secrete exosomes containing neurotrophic growth factors for healthy functioning of myelinization and axoplasmic flow [17]. The intracranial portion of the optic nerve is more sensitive to toxins than the retinal portion, as various toxins target the myelin sheath and axoplasmic proteins. In fact, this portion lacks the protective role of the blood-retinal barrier. Long-term stasis of the axoplasmic flow and imbalance of ion channels triggers the neuroinflammation and apoptosis mechanisms [15, 19].

Methanol is the most accessible industrial alcohol and disinfectant and the most common public health problem for optic nerve toxins in clinical practice, mostly due to fake alcohol production and drinks. When methanol is metabolized in the liver by the alcohol dehydrogenase enzyme, it is converted into formic acid and formaldehyde. Formic acid destroys oligodenrocytes and myelin sheath (demyelination) by causing metabolic acidosis. Formaldehyde disrupts adenosintriphosphate (ATP) synthesis by blocking the mitochondrial function and oxidative phosphorylation in the axons. Both metabolites block axoplasmic flows and destabilize the Na–K-ATPase and Ca/Calmodulin ion channels. Denatured proteins accumulate in axons and cause swelling in the ganglion cell complex (GCC). These swelling changes were also displayed in our study: by one of the features of OCTA. The accumulation of denatured proteins within axons leads to neuroinflammation. Neuroinflammation, disruption of ion channel balances, and blockage of ATP synthesis in mitochondria cause calcium ions to initiate apoptosis mechanisms in axons [4, 5, 20]. Methanol was the most common toxic substance (77.7%) encountered in our cohort study. Sildenafil is a vasoactive agent used in the treatment of erectile dysfunction. Its frequent use might lead to decreased optic nerve head blood flow and hypoxia. It is stated that non-arteritic ischemic optic neuropathy (NAION) occurring in male patients of a certain age may be associated with phofodiesterase inhibitors taken before [4, 21]. Carbon dioxide poisoning similarly leads to neural hypoxia. Hypoxia causes blockage of mitochondrial ATP synthesis and axoplasmic flow. It triggers axoplasmic swelling, neuroinflammation, and apoptosis [4]. Amiodarone is a potassium ion channel blocker drug used in the treatment of cardiac arrhythmias. Long-term use may cause disruption of ion channel balance in the optic nerve and blockage of axoplasmic flow. Changes in intra-axonal ion balances can lead to asymmetric neuroinflammation and apoptosis [4, 5].

Wharton’s jelly-derived mesenchymal stem cells (WJ-MSC) have a high paracrine effect and secrete exosomes into the chorioretinal microenvironment. Exosomes contain neurotrophic growth factors, various cytokines, and micro-RNA fragments. Neurotrophic growth factors accelerate ATP synthesis in mitochondria. The interleukin family and some other cytokines found in exosomes accelerate the digestion of denatured proteins and defective mitochondria found in the axoplasm. Autophagy and mitophagy help restore axoplasmic flow. Similar to the exosomes of oligodendrocytes, micro-RNA fragments contribute to remyelination. Some cytokines in exosomes prevent glial phagocytosis by immunomodulation and suppress neuroinflammation. All these factors cause inhibition of apoptosis mechanisms and increase the survival rate of axons and ganglion cells [6–10, 22, 23]. The subtenon space is a relatively avascular region and is a suitable culture medium for WJ-MSCs [22–24]. Retinal progenitor stem cells are administered subretinally or intravitreally due to their neuronal transformation properties. WJ-MSCs are used in clinical practice for secretory exosomes, not neuronal transformation [25–27]. Molecules smaller than 75 kD can pass through the scleral pores by passive diffusion. The passage of molecules larger than 75 kD through the sclera is possible with electrical/electromagnetic iontophoresis. Exosomes secreted by WJ-MSCs can pass from the sclera to the choroidal matrix passively or by electromagnetic iontophoresis. Growth factors in the exosome pass from the choroidal matrix to the subretinal space via tyrosine kinase receptors [28–37]. In our study, it was aimed that exosomes, not cells, reach the retina. For this reason, the subtenon region was preferred for WJ-MSCs.

Na/K-ATPase and Ca/Calmodulin ion channels in axons perform neurotransmission by providing neuronal polarization-depolarization and repolarization. Ion channels and intracellular and extracellular ion balances are disturbed in toxic optic neuropathies. Ion imbalances cause cells to switch to dormant phase or “off mode”. At this stage, neurons and axons are alive but unable to perform neurotransmission [19]. Repetitive electromagnetic stimulation provides rearrangement of ion channels and ion balances. rEMS accelerates neurotransmission and synaptic transmission with alternating current in neural tissues. rEMS increases the passage of large therapeutic molecules into neural cells by electromagnetic iontophoresis. rEMS accelerates blood flow and metabolism in neural tissues, increasing the intracellular elimination of glutamate and other metabolites. The ability of the axons to perform the polarization-depolarization-repolarization cycle allows the axons in “off mode” to be reactivated, that is, to switch to “on mode” [11–14, 34–37]. For these reasons, we investigated the effects of WJ-MSC and rEMS on toxic optic neuropathies as they are compatible with pathophysiology and mechanism of action.

BCVA improved significantly in all three groups. We observed that the combination of WJ-MSC and rEMS synergistically provides a more significant BCVA increase. We also observed the same synergistic effect on FPDI. GCC thickness decreased more in stem cell-treated groups 1 and 3 than non-stem cell-treated Group 2 (only rEMS applied). GCC thickness indicates the combined thickness of ganglion cells and retinal nerve fiber layer. GCC thickness may increase due to blockade of axoplasmic flow. This thickness may decrease with an improvement of axoplasmic flow or apoptosis of ganglion cells. We observed no significant improvement in visual acuity and visual field when GCC thickness was less than 60 μm, indicating atrophy. We believe that a GCC < 60 μm may be a sign of severe apoptosis and poor prognosis, according to our clinical observation. The decrease in GCC thickness in the stem cell groups was associated with significant improvement in BCVA and FPDI. In these groups, the higher rate of GCC over 60 μm can be explained by the improvement of the axoplasmic flow and less apoptosis rate. If there is no improvement in visual functions with the decrease in GCC thickness, we can think that this decrease is related to apoptosis. Another opinion; It may be that neurons surviving after a severe apoptosis increase the transition to ON mode with regenerative-restorative applications [38]. When pVEP p100 amplitudes were compared for all groups, we observed a similar increase in WJ-MSC when applied in Group 1 and Group 3. We found that this increase was less in the rEMS group alone. We believe that this situation is related to the increase in the number of reactive axons. All anatomical and functional data show that the combination of WJ-MSC and rEMS is synergistically more effective than individual applications of WJ-MSC or rEMS. We believe that the increase in intracellular transport of growth factors greater than 75 kD by electromagnetic iontophoresis also causes the combined treatment to be more effective [30–37].

The quantity of methanol consumed, hemodialysis application in the first 2 days, the use of bicarbonate to neutralize acidosis, the early use of ethanol as a competitive inhibitor of the enzyme alcohol-dehydrogenase, and of steroids with anti-edema and anti-inflammatory action, the serum level of vitamin B12 and the individual characteristics of the alcohol dehydrogenase enzyme in the liver influence the rate of toxicity and the relative extent of permanent damage to the optic nerve. The first emergency intervention was performed for all patients in specialized intensive care clinics participating in this study. The success rate was 77.8% in patients who received WJ-MSC and rEMS combination therapy in methanol intoxication. We think that 22.2% of unresponsive cases can be accounted for by the individual variables mentioned above. It is reported that progressive vision loss develops in the first 3 months when no treatment other than emergency intervention is applied in methanol intoxication. It is known that apoptosis continues rapidly and permanent axon and ganglion cell loss develop in the first 3 months. In toxic optic neuropathies, axons and ganglion cells are in the dormant/off mode before apoptosis. Incompatible axonal microenvironmental imbalance with vital conditions triggers apoptosis [4, 5, 20]. We believe that the increase in visual functions results from the neurotrophic growth factors, cytokines, and microRNA in WJ-MSC exosomes through a rearrangement of the microenvironmental balance. When the visual results were compared according to the groups, we observed that rEMS synergistically increased the efficacy of WJ-MSC. This can be explained by the fact that rEMS restores ion channels and ion balance, making axons suitable for impulse transmission [39–41].

Amiodarone is a cardiac antiarrhythmic that inhibits K channels. Long-term use of amiodarone can result in asymmetric toxic optic neuropathy. A patient who applied to our clinic complaining of a sudden decrease in vision in the left eye was consulted by the cardiology in terms of amiodarone intoxication. On examination, asymmetric TON was also detected in the right eye. We applied a combination of WJ-MSC and rEMS to the left eye and rEMS only to the right eye. We found a dramatic improvement in both eyes. Since methanol also disrupts axpolasmic flow, we detected central and centrocecal scotoma, while amiodarone only disrupts ion channels, we detected peripheral concentric scotoma. Significant improvement was also observed in the eye that was treated only with rEMS. This situation supports our hypothesis that rEMS acts by regulating the ion channel balance [24]. Sildenafil and carbon dioxide causes ischemic and hypoxic changes in the optic nerve. They can disrupt ATP synthesis, axoplasmic flow, and ion balance. At higher concentrations (> 10%), carbon dioxide may cause convulsions, coma and death. Corbon dioxide poisoning can occur in submarines and scuba divers when scrubbers aren’t functioning properly, as seen in our one case. Damages of toxins that cause hypoxia can also significantly improve with the early application of WJ-MSC and rEMS. We think that this effect is due to the fact that rEMS increases neural blood flow and the paracrine effect of WJ-MSC [39–41]. No systemic or local side effects due to WJ-MSC and/or rEMS applications were detected.

The study has some limitations. It has reported in the literature that the paracrine effects of WJ-MSC last for an average of 3 years. Longer follow-ups are needed to determine how long the effects will continue in our cases and whether additional applications will be needed. Determining how each exosome content specifically affects ganglion cells and axons is a separate topic of research. Another limitation is that the groups were small and mostly composed of methanol intoxication. Larger, multicenter studies will contribute to homogenizing the amount of poison, the duration of application, and individual characteristics. Large case series are also needed to examine the differences between treated and untreated eyes.

## Conclusion

Toxic optic neuropathies are emergent pathologies that can result in acute and permanent blindness. After poisoning with toxic substances, progressive apoptosis continues in optic nerve axons and ganglion cells. After the proper first systemic intervention, the WJ-MSC and rEMS combination seems very effective in the short-term period in cases with TON. To prevent permanent blindness, a combination of WJ-MSC and rEMS application as soon as possible may increase the chance of success in currently untreatable cases.

## Data Availability

The datasets generated during and/or analysed during the study are available from the corresponding author on reasonable request.

## Abbreviations

BCVA: Best corrected visual acuity
cGMP: Current good manufacturing practise
ETDRS: Early treatment of diabetic retinopathy study
FPDI: Fundus perimetry deviation index
GCC: Ganglion cell complex
GFs: Growth factors
OCTA: Optical coherence tomography angiography
rEMS: Repetitive electromagnetic stimulation
TON: Toxic optic neuropathy
WJ-MSC: Wharton’s jelly derived mesenchimal stem cell
VEP: Visual evoked potential
VF: Visual field
